# Microenvironmental Features Driving Immune Evasion in Myelodysplastic Syndromes and Acute Myeloid Leukemia

**DOI:** 10.3390/diseases10020033

**Published:** 2022-06-10

**Authors:** Georgios Petros Barakos, Eleftheria Hatzimichael

**Affiliations:** 1 First Department of Internal Medicine, General Hospital of Piraeus “Tzaneio”, 18536 Piraeus, Greece; bargeo46@gmail.com; 2 Department of Haematology, Faculty of Medicine, School of Health Sciences, University of Ioannina, 45500 Ioannina, Greece

**Keywords:** myelodysplastic syndromes, acute myeloid leukemia, immune evasion, bone marrow microenvironment

## Abstract

Bone marrow, besides the known functions of hematopoiesis, is an active organ of the immune system, functioning as a sanctuary for several mature immune cells. Moreover, evidence suggests that hematopoietic stem cells (the bone marrow’s functional unit) are capable of directly sensing and responding to an array of exogenous stimuli. This chronic immune stimulation is harmful to normal hematopoietic stem cells, while essential for the propagation of myeloid diseases, which show a dysregulated immune microenvironment. The bone marrow microenvironment in myelodysplastic syndromes (MDS) is characterized by chronic inflammatory activity and immune dysfunction, that drive excessive cellular death and through immune evasion assist in cancer cell expansion. Acute myeloid leukemia (AML) is another example of immune response failure, with features that augment immune evasion and suppression. In this review, we will outline some of the functions of the bone marrow with immunological significance and describe the alterations in the immune landscape of MDS and AML that drive disease progression.

## 1. Introduction

Bone marrow (BM) is a sophisticated organ, providing more than just passive hematopoiesis. BM supports the immune system through lymphopoiesis and myelopoiesis and is not an indifferent organ immune-wise, but rather a highly active one, with its components reacting to an array of immune and noxious stimuli, influencing the function of hematopoietic stem cells (HSCs) and hematopoietic niche cells [1]. Moreover, BM acts as a sanctuary for many immune cells, such as plasma cells and mature lymphoid cells [2]. Alterations in the niche components are associated with malignancies [3].

The BM immune landscape significantly changes with myeloid malignancies. In myelodysplastic syndromes (MDS) (a malignancy characterized by myelodysplasia and ineffective hematopoiesis [4], the HSC reactivity to environmental stimuli proves detrimental, as chronic inflammatory signaling drives MDS progression and normal HSC suppression [1,5,6]. Studies suggest that the difference between low-risk and high-risk diseases also manifests in their different immune microenvironments [7,8].

Acute myeloid leukemia (AML) is a highly heterogenous disease, which results in normal hematopoiesis failure, owing to immature myeloid cell proliferation [9]. The BM immune microenvironment is significantly dysregulated in AML and even though AML has been considered an immune responsive disease [10], AML cells are capable of evading and suppressing host immune responses [11].

In this review, we highlight the immune functions of the normal BM and further outline the dysregulations of the immune landscape that augment disease progression in MDS and AML (summarized in Table 1).

### 1.1. BM in Immunity and Inflammation

BM is the premier organ to produce different blood components during both steady-state and stress hematopoiesis. Hematopoietic stem and progenitor cells (HSPC) represent the functional unit; they self-renew and differentiate in a tightly regulated manner in heterogenous microenvironmental domains.

BM supports the immune system through myelopoiesis and lymphopoiesis, as well as a sanctuary for mature lymphoid cell types [2]. A topic of great interest is the function of BM besides that of a hematopoietic organ, having a key role in sustaining protective immune responses through plasma cell survival and immune memory maintenance [12,13], as well as being a dynamic entity capable of directly sensing and modulating its response to inflammatory signals [1]. Aberrancy in the components of the hematopoietic stem cell niche can induce and/ or sustain myeloid malignancy

### 1.2. Lymphopoiesis

HSCs differentiate into multipotent progenitors (MPPs) [14], which will differentiate into lymphoid and myeloid lineages through common lymphoid (CLPs) and common myeloid progenitors (CML), respectively [15]. Different HSC populations exhibit heterogenous potential for lymphoid commitment, suggesting the existence of intrinsic lymphoid bias [2], although HSC differentiation leans towards myelopoiesis with aging [16]. During lymphoid development, early T-cell progenitors migrate to the thymus, while B-cell progenitors maturate in the BM [2]. Distinct cellular niches guide B-cell developmental stages. Early-stage lymphopoiesis is associated with osteoblasts, osteoclasts, and CXCL12-abundant reticular (CAR) cells, whereas later development is regulated by interleukin (IL)-7 producing cells and sinusoidal endothelial cells [2]. IL-7 is pivotal in B-cell development [17].

### 1.3. Interplay between Lymphoid Lineage Cells and the BM

In addition to lymphopoiesis, BM harbors multiple mature lymphoid cells [2]. Naive T-/B-cells recirculate and populate the dendritic cell (DC) rich perisinusoidal space, forming a possible defense for blood-borne pathogens [18,19]. CD4+ and CD8+ memory T-cells can also be maintained in the BM after antigen experience. Whether memory T-cells are temporary inhabitants or a fraction of them permanently reside in the BM is an area of active research [20]. The current consensus is that a non-migratory CD69+ memory T cell subset exists, permanently localized in the BM [12], and a CD 69- memory T-cell subset exists in a state of equilibrium between the BM and the peripheral blood [12]. Regulatory T-cells (Tregs) are known immune mediators and have a crucial role in the success of allogeneic hematopoietic stem cell transplantation (allo-HSCT), as well as the progression of myeloid malignancies [3]. Tregs also regulate IL-7 production, and therefore, B-cell development [21]. T-cells also function in the context of inflammation by impairing HSC renewal through interferon (IFN)-γ [22,23] and inducing myelopoiesis through tumor necrosis factor (TNF)-a [24].

Plasma cells survive in the BM with stimuli from the microenvironment. These stimuli include the secretion of cytokines such as CXCL12, IL-6, B-cell activating factor (BAFF), and a proliferation-inducing ligand (APRIL) in specialized domains termed survival niches [25]. Ligation of B-cell maturation antigen (BCMA) by APRIL/ BAFF is a crucial step for plasma cell survival [26]. Mature cells of hematopoietic progeny and non-hematopoietic stromal cells contribute to the formation of the plasma cell niche [12,27].

Even though the BM is not a secondary lymphoid organ, some of its functions resemble one. The BM can conduct primary immune reactions (CD4+ and CD8+ T-cells) in response to blood-borne antigens [28,29], without forming organized B-/T-cell areas, which characterize secondary lymphoid organs, but rather aggregates of dendritic cells and T-cells [16]. Moreover, memory CD4+ T-cell responses, leading to cluster formation between major histocompatibility complex type II (MHC II) presenting cells and antigen-specific T-cells have been documented [30]. Histologically, lymphoid follicle-like structures, which are devoid of traditional B-/T-cell organized areas can expand during inflammation and autoimmunity [31].

### 1.4. Myelopoiesis

The BM microenvironment controls myeloid progenitor differentiation and distribution through opposing signals from different cell types [17,32]. For instance, Hérault et al. showed that granulocyte–macrophage progenitors (GMP) are scattered single cells during a steady state. However, GMPs expand and form clusters during regeneration and leukemia. G-CSF and IL-1 nurtured GMP cluster manifestation, whereas megakaryocyte-derived signals inhibited GMP promotion [33].

As stated above, the BM is not an organ that only produces immune cells, but also actively responds to harmful and inflammatory stimuli. HSCs are capable of directly sensing exogenous noxious and/or inflammatory signals such as cytokines, pathogen-associated molecular patterns (PAMPS), and damage-associated molecular patterns (DAMPS) with an inventory of intracellular and extracellular receptors. The resulting effects are HSPC mobilization and differentiation to augment immune cell production [1,34,35]. Moreover, it is known that a differentiation program can be preferred under specific conditions, as in the case of emergency myelopoiesis (sacrificing lymphopoiesis) during severe infection [34,36].

An array of different inflammatory cytokines such as IFN-a, IFN-γ, TNF-a, transforming growth factor (TGF)-β,IL-1, IL-6, macrophage-colony stimulating factor (M-CSF) secreted by hematopoietic progeny and non-hematopoietic cells affect HSC function and preferentially induce myeloid differentiation [1]. However, the effects of cytokines may be context-dependent and when maintained can have deleterious consequences on HSCs [1,37]. Toll-like receptors (TLRs), specifically TLR2 and TLR4, are expressed on HSPC and their activation by DAMPs and PAMPs results in myeloid differentiation [38]. Pattern recognition receptors (PRRs), such as TLRs and NOD-like receptors exist on hematopoietic and non-hematopoietic cells affecting HSC upon their activation. BM microenvironment cells of non-hematopoietic progeny such as endothelial cells, osteocytes, adipocytes, and neurons can modulate HSC functions in stressed conditions [1]. For instance, TLR-4 and NOD-like receptor 1/2 by microenvironment cells resulted in granulocyte-CSF(G-CSF) and induced a myeloid differentiation in HSCs [39,40]. Furthermore, HSCs and their BM niche respond to transient BM injury and inflammation by chemotherapy and radiotherapy to reinstate normal hematopoiesis [1].

In recent years, the concept of trained immunity of the innate immune system–analogous to the immunological memory of the adaptive immune system- has emerged, with its basis being epigenetic reprogramming of innate immune cells [41]. This trained immunity may come down to its effects on HSPCs giving another layer of complexity to the immune response [1,42].

### 1.5. HSCs and Allo-HSCT

HSCT is an effective method for treating hematologic malignancies; however, the myeloablative regimens enlisted are damaging to the recipients’ BM microenvironment, subsequently compromising donor HSCs’ function and transplant success (negative bystander effect) [3,43]. Transplanted HSC in order to reconstitute hematopoiesis must home and establish themselves in the damaged/reconstituting BM [44], with CXCL12 being critical (among many chemo-attractants) in mediating HSC engraftment [45,46]. Interestingly, donor HSCs can also influence the recipient’s BM microenvironment into augmenting their engraftment potential [44]. The elevated O2 levels (attributed to transplant pre-conditioning [47], metabolic switch to OXPHOS, and the resulting increase in ROS levels [48,49], are insults that transplanted HSC need to address in order to be functionally intact and balance between self-renewal and differentiation [44].

Immune reconstitution post-HSCT commences gradually at different phases, with the innate component being the first to function, with different transplantation procedures characterized by different neutrophil engraftment dates (umbilical cord HSCT being the slowest) [50,51], followed by NK and CD4+/CD8+ T-cells and lastly B-cell reconstitution [52]. Different transplantation modalities result in different immune reconstitution kinetics, with haploidentical HSCT having delayed immune reconstitution compared to matched-sibling donor transplantation [53]. Moreover, it is suggested that better immune recovery correlates with superior transplantation outcomes [54].

Now that some immune functions of the BM have been outlined, the contribution and perturbation of the immunological landscape of the BM in the context of MDS and AML will be discussed.

## 2. The Immune Landscape in MDS

**Figure 1:**
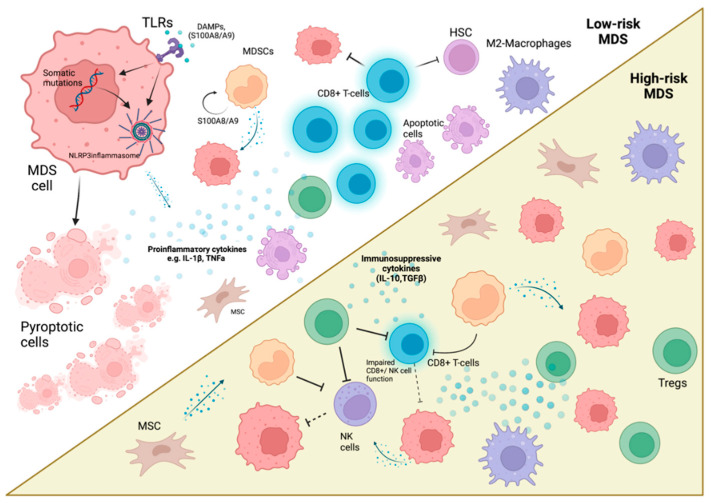
The immune landscape in low (top half) and high-risk (bottom half) MDS. NLRP3 inflammasome recruitment (by means of TLR signaling pathways (through DAMPs, PAMPs, S100A8/A9) and somatic mutations) by cancer cells is central in MDS pathogenesis driving proinflammatory cytokine production and pyroptosis. In low-risk disease, cytotoxic T-lymphocytes are increased, whereas Tregs are reduced. Cytotoxic T-cells suppress both malignant and normal HSCs further augmenting the cytopenic phenotype. High-risk disease is characterized by MDS clone expansion driven by immunosuppressive cytokine production, reduced efficiency of NK cells, and cytotoxic T-cells with concomitant expansion of Tregs and MDSCs. MSCs and M2-phenotype mac.

The Revised International Prognostic Scoring System (IPSS-R) is used to estimate the MDS patients’ risk of AML progression and overall survival (OS). In clinical settings, patients with an IPSS-R score of 3.5 or less represent a lower-risk MDS group (median survival; 5.9 years), whereas an IPSS-R score > 3.5 falls into the higher-risk MDS group (median survival; 1.5 years) [55]. The lower-risk disease is associated with an inflammatory microenvironment and increased cell death, in contrast to higher-risk disease, which is delineated by immunosuppression and clonal expansion (Figure 1) [7,56]. Herein, the role of inflammation and immunosuppression regarding their constituting cell types will be discussed.

### 2.1. The Inflammatory Microenvironment of MDS

Aberrant innate immune system activation leads to dysfunctional hematopoiesis and induces excessive cellular death; a hallmark of MDS [56]. Several factors, intrinsic and extrinsic of the malignant clone, contribute to the intricate inflammatory network.

#### 2.1.1. Predisposing Factors Driving Inflammation (Mutations, Aging/Chronic Immune Stimulation)

The genetic lesions of MDS are complex and dynamic and may enable a reciprocal mechanism in which gene mutations upregulate inflammation pathways and the inflammatory milieu contributes to genomic instability and mutagenesis [7]. Recurrent mutations entail spliceosome machinery, epigenetic and transcription regulation, DNA repair, signaling pathways, and the cohesin complex [4]. Spliceosome mutations such as SF3B1, SRSF2, U2AF1, and mutations in epigenetic regulators, e.g., TET2, and ASXL1, which drive clonal dominance and evolution in MDS [7], trigger innate immune signaling pathways and NRP3-inflammasome activation [7,57]. Haploinsufficiency of microRNAs (miR-145, miR-146a), as well as genes, in del (5q) MDS altered signaling intermediates, such as Toll-interleukin-1 receptor domain-containing adaptor protein (TIRAP) and TNF receptor factor-6 (TRAF6), stimulating TLR activation and cytokine (IL-6) production [58,59].

Aging represents a smoldering inflammatory process (“inflammaging”) [60] characterized by immunosenescence leading to malfunctioning adaptive immune responses [61,62]. As analyzed, HSCs are capable of directly sensing DAMPs/PAMPs through PRRs, skewing their differentiation program to myeloid lineages [16,38]. Such conditions create a fertile environment for mutated MDS clones to propagate [7]. Moreover, MDS is associated with a variety of autoimmune diseases, providing another example of chronic immune stimuli [8].

#### 2.1.2. Significant Signalling Pathways

Innate immune signaling is central in the pathogenesis of MDS. Genes related to immune signaling are overexpressed in more than 50% of MDS patients [63,64]. TLRs and their downstream intermediates (MyD88, IRAK1/4, TRAF6) are hyperactivated, whereas inhibitory regulators are repressed [5]. Sustained TLR activation is damaging to HSCs and their niche [65] and excessive TLR4 signaling results in genotoxicity, possible carcinogenesis [37], and cell death (along with TLR2) [66,67]. Alarmins S100A8 and S100A9 are of particular importance in MDS, because—via autocrine and paracrine actions—they drive TLR4 activation, NLR family pyrin domain-containing protein 3 (NLRP3) inflammasome assembly, and microenvironmental immunosuppression [6]. Pyroptosis—a form of programmed cell death—being driven by NLRP3 inflammasome activation, is responsible for cellular death in MDS, being a point of convergence for cell-intrinsic, (e.g., gene mutations) and cell-extrinsic, (e.g., DAMPs) pathogenesis mechanisms [57]. The NLRP3-pyroptosis axis functioned independently of specific gene class mutations [57]; however, pyroptotic cell death observed in erythroid progenitors was proportional to mutational allelic burden and complexity [6]. The cytopenic phenotype of MDS is further augmented by evidence of increased apoptotic signaling; activation of death receptor Fas–Fas ligand pathway through cytokine induction (like TNFa, IFN-γ), TNF receptor 1 and 2 (TNFR1 and 2) induced apoptosis and p38 mitogen-activated protein kinase (MAPK) apoptotic signalling [8].

#### 2.1.3. Cytokines

Aberrancy in the cytokine network has been observed in MDS patients by several investigators [8,56,68], with increased levels of inflammatory cytokines, such as TNFa, IFN-γ, TGF-β, IL-6, and IL-8, indicating abnormal inflammatory signaling and myeloid differentiation [8]. Various cell types can secrete cytokines in MDS, such as myeloid-derived suppressor cells (MDSCs) and MDS-derived myeloid cells [5]. Cytokine pressure from chronic immune stimulation has toxic effects on normal HSCs and may provide an evolutionary edge in MDS clones, driving their expansion [56]. In a first-of-its-kind meta-analysis, Xin et al. showed an increased level of inflammatory cytokines implicated in MDS pathogenesis and a cytokine profile changing along with the natural history of the disease [69]. Low-risk disease and its pyroptotic/ inflammatory microenvironment are associated with elevated levels of inflammatory cytokines (TNFa, IL-6, IFN-γ) [70] and type 1 cytokines (IL-1β, IL-7, IL-8), whereas high-risk disease has increased immunosuppressive cytokines (like IL-10), reflecting tumor immune escape [71]. Moreover, a positive correlation can be made between cytokine expression, clinical outcomes [56], and cytogenetic features [68].

### 2.2. Defective Cellular Immune Responses Result in MDS Progression

Cellular immune response dysregulation is another important immunological mechanism driving MDS pathogenesis, as it closely interacts with the coexisting inflammatory microenvironment and seems to have distinct features in different disease states [7].

#### 2.2.1. T-Lymphocytes in MDS

Low-risk MDS is characterized by increased numbers of cytotoxic (CD8+) T-cells and diminished counts of Tregs [72,73], contradicting the general finding of lymphopenia. The T-cell response of high-risk disease manifests itself with lower levels of CD8+ T-cells and higher numbers of Tregs [71,73].

CD8+ T-cells in low-risk MDS have the potential of suppressing both malignant and normal hematopoiesis in vitro, further augmenting cellular death in this disease state, because of the existence of epitopes on MDS HSCs that can activate cytotoxic T-cells [74]. In younger MDS patients, CD8+ autoreactivity seems to be correlated with reduced numbers of CD4+ helper T-cells [75]. Potential epitopes include Wilms tumor protein 1 (WT1), overexpressed in MDS with trisomy 8, cancer-testis antigen (CTA), proteinase 3, and MHC-I [8]. However, the impact of immunogenic neo-antigens is unexplored territory, with early studies pointing to the presence of a protective effect [7,76]. In high-risk MDS, failing immunosurveillance and anti-MDS function is the major characteristic of the adaptive immune response, possibly through immune checkpoint molecule upregulation [8], mainly cytotoxic T lymphocyte-associated protein 4 (CTLA4) and programmed cell death protein 1 (PD-1) [77]. MDS blasts in high-risk diseases overexpress PD-L1 in comparison to normal controls [78] and this can be further augmented by the effects of cytokines, such as TNF-a, and IFN-γ, which induce PD-1 and PD-L1 expression, on T-cells and MDS cells, respectively [79]. Interestingly, Sand et al. reported increased numbers of BM cytotoxic T-cells; however, they were accompanied by dysfunctional T-cell receptor (TCR) cytotoxicity [80].

Regarding helper T cells (Th); Th1/Th2 ratio imbalance might reflect CD4/CD8 imbalance [81]. Shao et al. reported no difference in Th1 in MDS patients and healthy controls, as well as between low versus high-risk diseases. Moreover, Th17 numbers were increased in MDS patients, with greater expansion in low-risk disease, denoting the auto-reactive, possible anti-tumor nature of the process [82,83], whereas Th22 cells were increased in high-risk disease, correlating with increased IL-6 and TNF-a [82].

Tregs are a subset of helper T-cells tasked with immune response modulation and immune tolerance [8]. As a result, reduced Treg functionality is associated with autoimmunity and increased Treg function with cancer cell immune evasion [84]. This is evident in low versus high disease states, where in low-risk disease, patients had diminished Treg numbers and function, in contrast to high-risk disease; increased Treg population and functionality, along with MDS clone expansion [81,85,86].

Apropos of the MDS mutational landscape, the presence of MDS founder mutations on the lymphoid lineage may lead to defective immune responses [7]. Furthermore, the TP53 mutated subset of MDS was associated with increased PD-L1 expression on MDS/secondary AML specimens, as well as ICOS High/PD-1neg Treg expansion, leading to an immunosuppressive microenvironment [87].

#### 2.2.2. Dendritic Cells in MDS

Dendritic cells are essential for tumor recognition, antigen presentation [88], and T-cell activation [89]. In MDS, dendritic cells have reduced T-cell activation potential and maturation defects—possibly stemming from the malignant clone [90]—along with a different cytokine profile between immature and mature monocytic dendritic cells (high IL-10 and low IL-12) [91]. Moreover, the high-risk disease has been shown to have reduced numbers of myeloid and plasmacytoid precursor dendritic cells [92].

#### 2.2.3. Natural Killer Cells in MDS

Natural killer cells (NK cells) are a lymphoid subset of innate immunity and their aberration in MDS represents another aspect of defective immune surveillance. Studies have shown low NK cell numbers and impaired NK cell performance [93,94]. Cytotoxic impairment was especially evident in high-risk MDS, which correlated with advanced disease burden (excess blasts, high IPSS score, cytogenetics) [95,96]. Shared genetic lesions with the malignant clone and NK populations might explain an intrinsic defect in the functionality of NK cells [97].

#### 2.2.4. Myeloid-Derived Suppressor Cells in MDS

Myeloid-derived suppressor cells (MDSCs) are a heterogeneous population of the myeloid lineage, which expand in pathological conditions such as cancer [98]. They have the ability to suppress non-myeloid immune cell activity, such as T-cell, B-cell, and NK-cells [99,100] and modulate macrophage cytokine production [101]. They can also assist Treg expansion through IL-10 and TGF-β secretion [102]. In MDS, high counts of MDSCs were reported in the BM [103] and peripheral blood of patients [104]. MDSC expansion can be induced by a number of pro-inflammatory cytokines such as IL-6, IL-10, IL-1β, and IFN-γ [102]. Alarmin S100A9 seems to drive MDSC expansion in MDS [103]. Moreover, concomitant age-related myeloid skewing, senescence, and “inflammaging” are involved in age-related MDSC increase [100,105]. In high-risk diseases, Kittang et al. [106] showed MDSC and Treg associated expansion, assisting in immune evasion and cancer progression. MDSCs also play a role in ineffective hematopoiesis [103]. Interestingly, MDSCs in MDS patients seem to evolve from a non-MDS clone, as they did not harbor the same mutations [103].

#### 2.2.5. Macrophages in MDS

Macrophages come from monocytic differentiation and exhibit a diverse set of biological roles [107]. Defective macrophage function may result from reduced CD206 and signal regulatory protein alpha (SIRPa) expression, as well as increased inducible nitric oxide synthase (iNOS), which aids cancer progression through NO production [108]. INOS+ macrophages were associated with low-risk disease [109]. Macrophages are divided into M1 and M2 subtypes, with M1 having antitumor effects [110]. M2 subtype, but not M1 expansion was observed in MDS, suggesting a further reduced antitumor effect [111].

#### 2.2.6. Mesenchymal Stem Cells in MDS

MSCs are an essential non-hematopoietic subset for hematopoiesis. Although inconclusive, MSCs might have a role in MDS initiation and maintenance, through pro-inflammatory signaling contributing to immune suppression and mutagenesis [7,8]. MSCs exhibit an age-related wane in functionality [112]. This is accompanied by the fact that MDS/AML-derived MSCs have functional deficits, as well as chromosomal aberrations different from the malignant clone [7]. Meydouf et al. elegantly showed the influence of MDS HSCs on their microenvironment with their ability to induce gene expression changes in healthy MSCs giving them an MDS-like phenotype [113]. MDS-MSCs produce factors such as S100A8/9 and immunomodulators that enable MDS clone expansion [114,115,116]. Interestingly, MSCs seem to exhibit different characteristics between risk groups [8]. Low-risk disease MSCs showed reduced DC maturation and differentiation efficiency, whereas high-risk disease MSCs demonstrated immunosuppressive properties, higher TGF-β production, apoptosis, and Treg induction [117,118].

## 3. Immune Evasion in Acute Myeloid Leukemia

**Figure 2:**
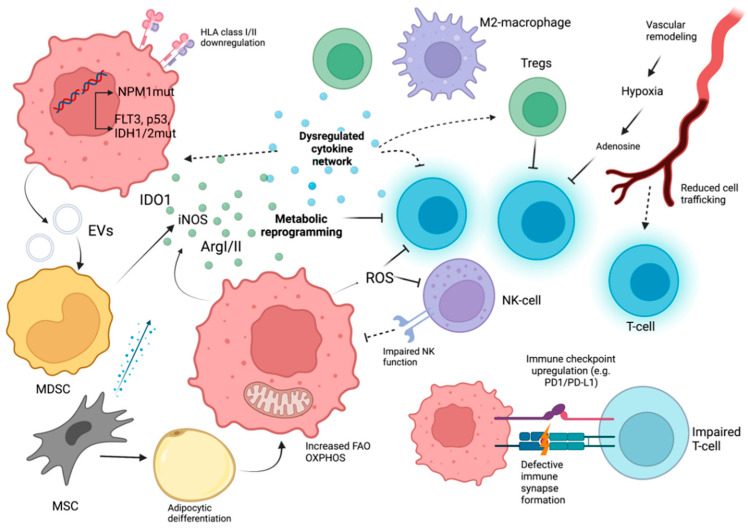
The immune landscape in AML. A complex network of AML cell-extrinsic and intrinsic factors characterize the immune microenvironment of AML. AML-related somatic mutations are implicated in immune alterations (with NPM1mut involved in effective T-cell immune responses). HLA molecule downregulation and defective antigen presentation are also recruited. Altered cytokine balance also assists AML cell expansion, Treg function, and immune suppression. A number of metabolic factors (IDO1/iNOS/ArgII elevation, increased fatty acid oxidation, and oxidative phosphorylation) contribute to immunosuppression. MDSC expansion (possibly through MUC1 containing extracellular vesicles) and M2-macrophages also augment AML immune escape. Immune checkpoint molecule overexpression on AML and CD8+ T-cells (e.g., PD-1/PD-L1, TIM-3/galectin-9), poor immune synapse function, and impaired NK –cell function (through NK-cell receptor downregulation, defective NK degranulation, and perforin binding). BM vascular remodeling impedes anti-AML immune responses through reduced cell migration and hypoxia induction.

From an immunological perspective, AML blasts and leukemic stem cells (LSCs) recruit several intrinsic and extrinsic immune evasion mechanisms, that along with an assisting BM microenvironment remodeling drive disease progression (Figure 2).

### 3.1. Cell Intrinsic Factors in AML

Intrinsic characteristics of the heterogenous AML population form the foundation of the defective immune response [11]. The mutational landscape of AML is characterized by an average of 13 mutations in coding genes per patient, with 5 genes being recurrently mutated [119]. Genetic stratification systems assess the impact of these recurrent mutations on survival [120]; however, the reciprocity between genetic lesions and the immune dysregulation of AML is less characterized (reviewed in [121]). Herein we present the immunological consequences of the commonest mutations. Evidence suggests that nucleophosmin1 (NPM1) mutations, mostly associated with favorable prognosis, have the potential to elicit T-cell responses [121]. Fms-like tyrosine kinase 3 (FLT3) mutations, i.e., internal tandem duplication (ITD) and tyrosine kinase domain (TKD) have been correlated with immune response alterations in AML [121]. Clinical studies have shown that ITD was associated with high populations of DCs, pre-DCs, and Tregs [122]. Moreover, immunological extensions of FLT3 inhibitor therapy have been reported [123]. The effects of mutant IDH1/2 and the oncometabolite R-2-hydroxyglutarate (R2-HG) on the immune system have been studied in gliomas extensively, with evidence of T-cell suppression and reduced innate immune system function [121]. In the setting of AML, R-2-HG was associated with Treg development [39]. In addition to functioning as the guardian of the genome, TP-53 may have tumor-suppressing properties stemming from the regulation of immune responses, and p53 mutations may reduce immunosurveillance and create a cancer-promoting immunosuppressing microenvironment [121] (in parallel with MDS [87]).

Immunoediting properties of leukemic blasts, such as downregulation of mismatched HLAs in AML blasts [124,125,126] and epigenetic downregulation of class II HLAs [127,128], have been observed in the context of alloHSCT (different donor settings). This gives a fitness advantage in AML blasts and contributes to Graft versus Leukemia (GvL) evasion and post-HSCT relapse [129]. Impaired antigen presentation via HLA class II may also play a role in immune evasion [130]. Moreover, immunosuppressive class I molecule HLA-G may contribute to the immunosuppressive microenvironment through DC, T-cell, NK cell, and monocyte inhibition [129]; however, these findings are not solid [131].

As described in the above section, NLRP3 inflammasome activation is central in the pathophysiology of MDS. On the contrary, the role of NLRP3 inflammasome in the pathogenesis of AML is not well documented. Zhong et al. showed that AML cells overexpressed NLRP3 inflammasome components and the NRLP3 inflammasome assisted in AML propagation in an IL-1β dependent manner [132]. The same authors also demonstrated immunological implications of the NLRP3 inflammasome, which is associated with an aryl-hydrocarbon receptor (AHR) contributed to AML T helper cell abnormalities [133].

Immune checkpoint molecules and secretion of factors with immunomodulating capacities will be presented in later sections.

### 3.2. Cytokines in AML

Cytokine network aberrations have been reported mainly in primary AML and dysregulation between pro-inflammatory and anti-inflammatory cytokines provides a fertile tumor-promoting microenvironment [134]. Regarding pro-inflammatory cytokines, IL-1β is the premier cytokine studied in AML and is known to assist in AML cell growth [134]. IL-6 and TNF-a are also implicated in leukemic growth, survival, and drug resistance [134]. IFN-γ effects on AML cells maybe be determined by the microenvironmental cytokine network [135]. IL-2 produces two-sided effects by supporting lymphocyte function, however, also promotes Treg expansion and therefore immunosuppression [129]. On the other hand, TGF-β was shown to inhibit AML growth [136] and lower levels of TGF-β were found in AML patients [137,138] indicating that diminished TGF-β effects promote AML survival [134]. IL-10 may impede AML cell growth [134] and has been shown to be elevated in AML patients [138]; however, its impact on survival is unclear [138,139] and may also depend on the local cytokine/ cellular microenvironment [134]. Interestingly, anti-inflammatory cytokine IL-35 is elevated in AML and correlated with Treg expansion (IL-35 is secreted primarily by Tregs), as well as CD4+ and CD8+ inhibition [134].

### 3.3. Metabolic, Soluble, and Vascular Factors with Immunological Significance in AML

In addition to cytokines, several soluble factors and metabolites contribute to the immunosuppressive microenvironment of AML. AML blasts, MSCs and MDSCs have the capacity of secreting indoleamine 2,3- dioxygenase 1 (IDO1) [140]. IDO1 (a catabolic enzyme of tryptophan metabolism) activity leads to local tryptophan depletion and accumulation of noxious tryptophan metabolites, resulting in Treg expansion and effector T-cell inhibition and apoptosis [140,141], as well as dismal prognosis [142]. Increased arginase II levels associate with inhibition of T-cells and hematopoietic progenitors and macrophage skewing towards M2-phenotype [143]. In addition, iNOS upregulation by AML cells is also associated with suppressed T-cell proliferation and NKT cell numbers, along with increased Tregs [144].

Evidence suggests that AML-induced metabolic remodeling, inhibitory molecule production, (e.g., ROS), and competition for nutrients are associated with immune suppression [145]. MSCs are skewed towards adipocyte differentiation in AML and directly provide metabolic support to AML blasts [146] by enhancing fatty acid oxidation [147,148]. Fatty acids and FAO impede T-cell effector functions and aid in Treg and M2 macrophage generation [149]. MSCs also assist in AML OXPHOS through mitochondrial transfer mediated by AML NOX2 activity [150]. High amounts of ROS generated by NOX and OXPHOS activity [151] assist in T and NK cell suppression via PARP-1 dependent apoptosis [152].

Abnormal remodeling of the vascular component in the AML niche translates into further immune dysregulation [140]. Firstly, poor BM perfusion and hypoxia result in T and NK cell suppression through adenosine [153]. High extracellular amounts of ATP in AML lead to adenosine production through CD73 and CD39 enzymes on AML cells, Tregs, and MDSCs [154]. NO-mediated vascular remodeling promotes the hypoxic niche [155]. Moreover, vascular remodeling obstructs T-cell trafficking [140] and impaired immune cell adhesion to the endothelium has been reported, due to elevated E-selectin [156].

### 3.4. Cellular Immune Response Dysregulation

#### 3.4.1. T-Cells in AML

No consensus has been made regarding T-cell distribution and function in AML, possibly because of disease and patient heterogeneity along with different approaches in T-cell investigation [157]. Le Dieu et al. reported increased numbers of circulating T-lymphocytes in AML patients (in contrast to healthy controls), with a marked increase in a less clonal CD8+ population compared to CD4+ T-cells. Interestingly, BM T-cells were comparable between the two groups [158]. Increased CD8+ T-cells have also been reported by other groups [159]. Moreover, the T-cell activation signature was also elevated [158]. However, some studies demonstrated no increase in lymphocyte numbers between AML and controls but exhibited reduced CD4/CD8 ratios [160,161]. Nevertheless, higher BM lymphocytes with predominant T-cells were associated with superior OS [162] and strong lymphocytic increase post chemotherapy with decreased relapse risk [163].

Data suggest that T-cell dysfunction/exhaustion is present at diagnosis and T-cell functional status may be in a dynamic relationship with disease phase and treatment [157]. Le Dieu et al. showed that AML T-cells had different transcription profiles compared to controls, with some distinctively expressed genes contributing to cytoskeleton formation [158]. Subsequently, they showed an in vitro T-cell impairment in immune synapse formation [158]. A significant immune evasion mechanism is the expression of immune checkpoint ligands by leukemic blasts [128,164]. Human studies also demonstrated that T-cells from AML (co-) expressed higher levels of immune checkpoint molecules (PD-1 and TIM3, PD-1 and LAG3) compared to controls and their expression frequency increased with disease progression [165]. PD-L1 levels on AML cells were wide-ranging across different studies (from 18% to more than 50%) and PD-L1 expression was associated with acute monocytic leukemia and correlated with dismal prognosis [10]. TIM3 and its ligand galectin-9 (expressed on AML cells) induce β-catenin and NF-κB mediated self-renewal and NK-/T-cell suppression [166]. Furthermore, elevated mRNA levels of CTLA4 and LAG3 were found in T-cells correlating with inferior prognosis [167]. Similarly, several studies have shown that immune checkpoint expression in CD8+ is associated with T-cell dysfunction and leukemia relapse [10]. A study by Schnorfeil et al. demonstrated that cytokine production and proliferation of T-cells from AML patients with different diseases are unimpaired (in addition to reduced CD4+ IFN-γ production), with PD-1 overexpression representing differentiated effector T-cells, not exhaustion [168]. AML CD8+ T-cell cytokine production may not diverge from normal controls, even though exhausted subsets have been identified as stated [10]. Moreover, transcriptional signature reversibility of CD8+ cells in induction chemotherapy responders was demonstrated [165,169,170,171,172]. Remarkably, Radpour et al. suggested that in favorable-risk AML the BM CD8+ T-cells assisted in LSC expansion. Transcriptomic analysis of these CD8+ T-cells revealed epigenetic alterations affecting T-cell function [173]. On the contrary, high-risk disease LSC growth was a cell-intrinsic event and T-cell independent [173].

Regarding T helper cell function, Th1 cell numbers and functional capacity (IFN-γ production) may be decreased [168] with Th17 increase along with elevated IL-17 secretion associated with diminished Th1 and IFN-γ levels [108,174].

Treg numbers and function might be upregulated, as shown by several studies. In one study, peripheral and BM Tregs were increased compared to controls and BM Tregs exhibited higher immunosuppressive capabilities than their peripheral counterparts [175]. AML blasts that express inducible T-cell-co- stimulator ligand (ICOSL), promote Treg proliferation that contributes to disease maintenance via IL-10 secretion [176]. Furthermore, CXCL12/ CXCR4 may be crucial for Treg accumulation in the BM [177]. Variable levels of Tregs during treatment have been demonstrated [178,179]. Murine models and clinical studies have shown higher Treg levels at diagnosis contribute to disease propagation and inferior outcomes [175]. Contradictive results were demonstrated by a retrospective study; higher Tregs levels during lymphocytic recovery after induction were associated with improved response and survival [180].

#### 3.4.2. Dendritic Cells in AML

A study in AML patients by Derolf et al. demonstrated that DCs were diminished or even absent in AML BM at diagnosis compared to control, with DC regeneration observed in CR patients (even though plasmacytoid dendritic cells (pDC) were still reduced) [181]. The authors found no correlation between DC levels and survival [181]. Xiao et al. showed a significant reduction of plasmacytoid dendritic cells (pDC) in AML patients, as well as blast: pDC ratio correlated with positive measurable residual disease and poor outcome [182]. On the contrary, elevated DC proportions of peripheral blood mononuclear cells have been reported accompanied by immunosuppressive characteristics [122], an effect reversed with a TLR7/8 agonist [183,184]. Additionally, tissue forming-pDC (TF-pDCs) positive patients had inferior prognosis [185].

#### 3.4.3. NK Cells in AML

NK cell impairment in AML further contributes to the immune-evasive nature of the disease. Experimental studies have shown that TGF-β and IL-10 impede NK activity [186]. Tregs are capable of inhibiting NK activity through a TGF-β mediated mechanism [187]. NK dysfunction in AML-derived NK cells is a result of poor expression of natural cytotoxic receptors (NCR) [188]. The functional and phenotypic alterations of NK cells were reversible in patients who achieved remission, in contrast to treatment non-responders [189]. Moreover, the activating receptor NKG2D is poorly expressed in AML [186,190,191]. AML cells downregulated NCR-ligand expression leading to NK cytotoxicity evasion [188], with epigenetic mechanisms contributing to this [188,192,193,194].AML blasts can also avoid NK-mediated cell-lysis through weakened perforin binding [195].

#### 3.4.4. MDSCs in AML

Similarly, MDSCs in AML are an important medium in T- and NK- cell immunosuppression. MDSC numbers in AML patients are elevated in BM and PB, with MDSC increase correlating with MRD positivity [196]. MDSC proliferation can be induced by AML blasts themselves, via oncoprotein MUC1 containing extracellular vesicle production (EV). MUC1 increases cMYC expression in EVs resulting in MDSC growth [196]. The Akt/mTOR pathway is also implicated in the AML-EV mediated transition of monocytes to MDSCs [197]. MDSC suppressing mechanisms include V-domain Ig suppressor of T-cell activation (VISTA), PD-L1, IDO1, TGF-β, IL-10, ROS, peroxynitrite, PGE2, and exosomes [198].

#### 3.4.5. Macrophages in AML

Macrophages demonstrate endogenous plasticity capable of driving cell polarization under tissue-specific conditions [199] and in AML leukemia blasts have the ability to reprogram macrophages towards leukemia promoting the M2 phenotype [199]. AML blasts can induce M2 polarization via arginase II production [143,200]. Furthermore, higher numbers of M2-like macrophages were found in AML patients’ BM compared to controls and AML cells could induce an M2-like phenotype, possibly through dependence on transcription factor Gfi-1 [201]. Epigenetic and miRNA contributors may also be involved in M1 polarization impairment [200].

#### 3.4.6. MSCs in AML

AML-derived MSCs show higher immunosuppressive behavior like lymphocyte suppression and reduced proinflammatory cytokine production, compared to normal controls [139]. MSCs in AML diagnosis demonstrate higher VEGFA, CXCL12, PGE2, IDO1, IL-1β, IL-6, and IL-32, with reduced IL-10 compared to MSCs in AML relapse [116]. AML patient-derived MSCs exhibit Treg induction and IDO1 upregulation abilities [202]. Moreover, MSCs protected AML blasts from NK killing in co-culture systems [203], with TLR4 playing a role [204]. MSCs also inhibit NK expansion through IDO and PGE2 [204].

It should also be noted that the BM microenvironment assists in cancer resistance to chemotherapy via various different mechanisms (soluble factor-mediated or cell-adhesion mediated) from endothelial cells, MSCs, and osteoblasts [205]. Activated endothelial cells for example are important factors that drive leukemia relapse. AML-induced endothelial activations result in AML cell proliferation and cytarabine resistance [206]. Age-associated BM alterations have also been described in mice to affect young HSC engraftment and T-cell production when they were transplanted to old recipients compared to young [207].

## 4. Conclusions

Evidence suggests that the BM can participate in sophisticated immune responses since it harbors the appropriate immune cell types. The HSCs themselves have proper adaptation mechanisms to exogenous immune and inflammatory factors, which may become deleterious when sustained in time, leading to HSC malfunction, myeloid bias, and propagation of malignancies. MDS is a neoplastic disease, in which—as outlined above—chronic microenvironmental inflammatory signaling leads to neoplastic clone progression. Current studies indicate a dichotomy between low- and high-risk MDS immune milieu low-risk MDS manifests with an inflammatory cytopenic phenotype, whereas high-risk MDS clones take advantage of the suppression and reduction of various effector cell types and the upregulation of immunosuppressive cells. Similar to high-risk MDS, AML also recruits an inventory of mechanisms aimed at immune suppression and evasion of anti-leukemic cytotoxic cells. Of course, in reality, these diseases are much more complicated, as shown by Guo et al. who reported a highly heterogenous AML immune landscape with newly defined exotic immune cell subsets [208]. Our review only outlines the increasingly complex immunological landscape of these myeloid malignancies, provided by an ever-expanding number of studies. Nonetheless, the understanding of the immune dysregulation in MDS and AML will pave the way for the development of novel treatments that are so much needed for this patient population.

## Figures and Tables

**Table 1:** Summarizing evidence regarding immunology in low-risk/high-risk MDS/AML. MDSC: myeloid-derived suppressor cells, MSCs: mesenchymal stem cells.

	Low-Risk MDS	High-Risk MDS	AML
Cell intrinsic factors	Increased NLRP3-inflammasome activation and Pyroptosis Apoptosis induction	NLRP3 inflammasome activation	Downregulation of mismatched HLAs, class II HLAs (in alloHSCT) Impaired antigen presentation
Cytokines/Metabolites	Increased proinflammatory cytokine production (TNFa, IL-1β, etc.)	Immunosuppressive cytokine production (IL-10)	Increased Il-1β/ΙL-6/TNFa/IL-35 Decreased TGFβ Increased immunosuppressive metabolic factors (IDO1, ArgII, iNOS, FAO, etc.) Vascular remodeling with immunosuppressive sequelae
T-lymphocytes	Increased CD8+ lymphocytes with autoreactive potential Increased Th17 helper cell numbers Decreased Treg number	Diminished and dysfunctional CD8+ lymphocytes Increased immune checkpoint molecule expression Increased Th22 helper cells Tregs with increased number and functionality	Reduced CD8+ lymphocyte function/Increased immune checkpoint molecule expression Increased Treg numbers with immunosuppressive properties
NK-lymphocytes		Decreased NK-cell numbers with reduced function	NK-cell impairement
MDSCs	MDSC expansion	MDSC expansion correlating with Treg increase	MDSC expansion
Macrophages	M2 macrophage expansion INOS+ subtype increase	M2 macrophage expansion	M2 macrophage expansion
MSCs	Reduced differentiation potential	Increased with immunosuppressive properties	Increased immunosuppressive behavior

## Data Availability

Not applicable.
